# Plaque Inhibitory Effect of Hyaluronan-Containing Mouthwash in a 4-Day Non-Brushing Model

**DOI:** 10.3290/j.ohpd.a43936

**Published:** 2020-04-01

**Authors:** Begüm Gizligoz, Gizem Ince Kuka, Ogul Leman Tunar, Ebru Ozkan Karaca, Hare Gursoy, Bahar Kuru

**Affiliations:** a Dentist, Private Practice, Istanbul, Turkey. Performed the experiments in partial fulfilment of requirements for a degree.; b Assistant Professor, Department of Periodontology, Yeditepe University Faculty of Dentistry, Istanbul, Turkey. Contributed to the idea, hypothesis and conduction of the experiment.; c Assistant Professor, Department of Periodontology, Yeditepe University Faculty of Dentistry, Istanbul, Turkey. Contributed to the experimental design and discussion.; d Assistant Professor, Department of Periodontology, Yeditepe University Faculty of Dentistry, Istanbul, Turkey. Contributed to manuscript preparation.; e Associate Professor, Department of Periodontology, Yeditepe University Faculty of Dentistry, Istanbul, Turkey. Contributed to manuscript preparation.; f Professor, Department of Periodontology, Yeditepe University Faculty of Dentistry, Istanbul, Turkey. Contributed substantially to discussion and manuscript proofreading.

**Keywords:** chlorhexidine, hyaluronic acid, mouthwashes, gingivitis, taste perception

## Abstract

**Purpose:**

Despite being the gold standard antiplaque agent, chlorhexidine (CHX) has many adverse effects that make scientists search for new agents to combat biofilms as effective as CHX. Hyaluronan, also known as hyaluronic acid (HA), is a natural polysaccharide with anti-inflammatory, antioxidant and bacteriostatic properties. The objectives were to evaluate the plaque inhibitory, and anti-inflammatory effects of HA mouthwash compared to CHX and distilled water (DW) in a 4-day non-brushing model together with the participants’ preference to the used products.

**Materials and Methods:**

Thirty-three systemically and periodontally healthy subjects were included in this randomised, double-blinded, crossover clinical study. Subjects were randomly assigned into three treatment-sequence groups to use three mouthwashes one after another, in three different time periods. After professional prophylaxis at day 1, subjects refrained from all oral hygiene measures and used mouthwashes that were individually allocated to them. On day 5, scoring of plaque index (PI) according to Turetsky modification of Quigley Hein Index system, modified gingival index (MGI) and measurement of gingival crevice fluid (GCF) volume were performed. Treatment satisfaction questionnaire form was given at the end of each experimental period.

**Results:**

CHX showed statistically significant reduction in PI followed by HA (p = 0.048). No statistically significant differences were detected between HA and CHX in terms of MGI and GCF volume. For HA, subjects reported significantly better taste, less sensitivity, burning sensation, mouth dryness and numbness perception compared to CHX and DW.

**Conclusions:**

CHX revealed the best plaque inhibition closely followed by HA. Early gingival inflammatory changes were found similar for CHX and HA. Furthermore, HA was well accepted with better perceptions than CHX and DW.

Recognising that dental biofilm is the main local cause of gingivitis and periodontitis, prevention and treatment of these conditions are still based on self-performed plaque control and on professional removal of microbial deposits as well as reduction of microbial load at regular intervals.^25^ Mechanical biofilm control with toothbrushes and interdental devices disturb and remove biofilm and is the main means for a proper oral hygiene.

In the literature, mechanical devices demonstrate their efficacy in biofilm and gingivitis control.^35^ However, mechanical means alone may not be enough in a large proportion of the population for the prevention or the reactivation of the diseases.^9^ Therefore, the adjunctive use of chemical biofilm control has emerged.^41^ Chemical agents interfere with the stage cycles of biofilm formation. Conceptually, antiplaque agents can be used (i) to interfere with the adhesion of oral bacteria to surfaces and prevent biofilm formation; (ii) to interfere with coaggregation mechanisms or to affect bacterial vitality, which that prevent further growth of colonies; or (iii) to remove or to disrupt existing biofilms.^3^ Although chlorhexidine is a gold standard, there are many reported adverse events for this agent such as staining, increase in calculus formation, taste alterations, gingival desquamations, hypersensitivity reaction, delayed wound healing, uni- or bilateral tumefaction and short-term usage.^11,23,44^ Research continues for new adjunctive agents to combat biofilms efficiently and safely. Hyaluronan, also known as hyaluronic acid (HA), is one of the chemical agents recently under investigation and is a high molecular weight non-sulphated polysaccharide.^4,18^ It is biocompatible, non-immunogenic, biodegradable, viscoelastic that make it a preferable biomaterial for medical and pharmaceutical applications without adverse events.^5^ HA enhances regeneration, stimulates osteoinduction and involves in osseointegration.^2,14,16^ It also has inductive wound healing and antiadhesive effects when used topically.^29,33,45^ It is characterised by anti-inflammatory properties and also has bacteriostatic and antioxidant effects.^39^ The question remains whether HA has a plaque inhibitory effect, based on the aforementioned-properties. When the periodontal literature is reviewed, there are only a few studies evaluating its efficacy, both in vitro and in vivo. In vitro studies have shown that HA impedes bacterial growth, interferes with bacterial structure and morphology.^32,34,36^ In terms of clinical studies, it has been found that HA reduces plaque accumulation and inhibits gingival inflammation.^13,32,34^ However, there is still paucity of information in the literature on this area.

Therefore, the aim of this study is to evaluate the plaque inhibitory effect of HA mouthwash in comparison with chlorhexidine and distilled water (DW), in a 4-day non-brushing model using a double-blinded, randomised controlled study design.

## Materials and Methods

### Study Population

The study population was selected among the systemically and periodontally healthy dental students of Yeditepe University Dental School aged between 23 and 25 years. The Ethical Committee of Yeditepe University School of Medicine approved the study protocol with the origin in the Declaration of Helsinki (Decision No: 652/2016). This study has been registered to Thai Clinical Trial Registry (Identification number: TCTR 20181114005). The inclusion criteria were as follows: presence of at least 24 natural teeth (excluding third molars); healthy or early gingivitis subjects^10^; no fixed or removable prostheses and orthodontic appliances; no predisposing oral factors causing local irritation and plaque retention; no presence of systemic diseases; no lactation or pregnancy; no history of drug abuse; no medications; no use of systemic or topical oral antimicrobial therapy in the previous 3 months; not a current smoker or smoker over the past year. Subjects fulfilling the inclusion criteria and willing to actively participate in this study were asked to sign a consent form prior to the study procedures.

### Sample Size Calculation

Plaque index (PI) was chosen as the primary outcome variable and size estimation was performed based on a previous study.^12^ Calculations were performed with G* Power and Sample Size Program (www.powerandsamplesize.com/Copyright 2013–2018 HyLown Consulting LLC, Atlanta, GA, USA). Based on the data from the aforementioned-study of a 4-day non-brushing model, the calculated effect size by the program was 0.5921 (standard deviation of 0.7 and α error of 0.05 to obtain 80% power). Under this assumption, 33 subjects were taken for this crossover study with any possible dropouts (10%) to be allocated into three different sequences of treatments. Gingival index (GI) and gingival crevicular fluid (GCF) volume were evaluated as the secondary outcome variables.

### Treatment Products

Treatment products used in this study were as follows: chlorhexidine-containing mouthwash (CHX) was the positive control product and the commercial brand is known as Klorhex. It contains 0.2% chlorhexidine gluconate as an active ingredient, water, 2% glycerine as an inactive ingredient and 0.2% lemon scent and 0.02% mint scents as flavour. Hyaluronic acid-containing mouthwash (HA) was the test product and the commercial brand is known as Gengigel Hydrogel. It contains 0.025% of HA and 7.5% xylitol as active ingredients: water, cellulose gum, alcohol, PEG40 hydrogenated castor oil, polyvinyl alcohol, polycarbophil, 2,4 dichlorobenzyl alcohol and sodium as non-medicinal ingredients and a blend of essential oils (citromint) as flavour. DW was used as the negative control.

### Treatment Groups

**Fig 1:**
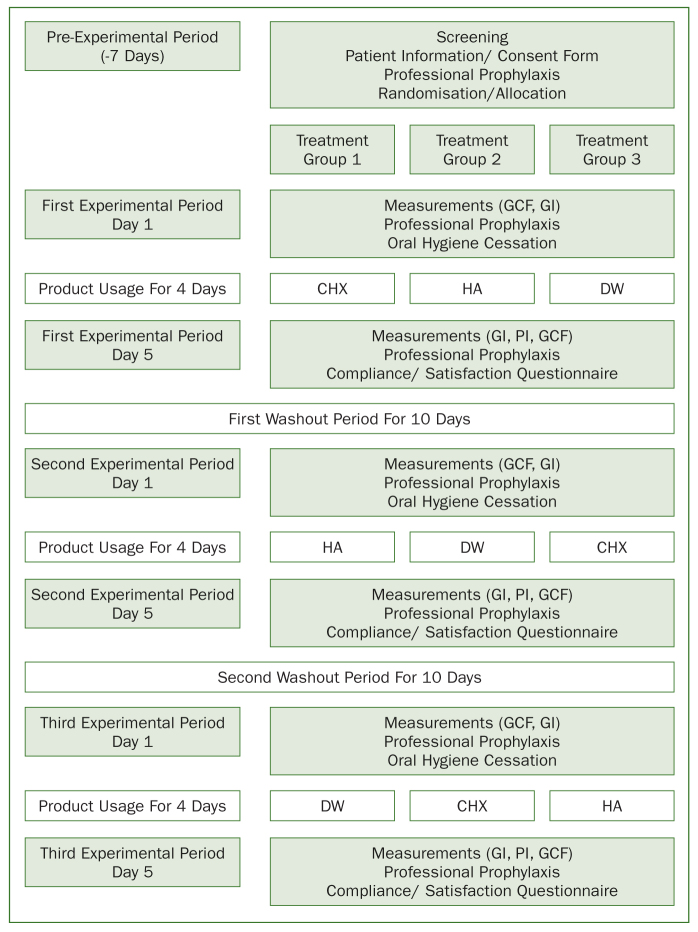
The flow chart of the study.

Subjects included in this study were coded with numbers as 1–33 and equally randomised into three treatment-sequence groups (n = 11 for each) by a computer generated-program (www.randomizer.org, Copyright © 1997–2018 by Geoffrey C Urbaniak and Scott Plous), by HG to use three different treatment mouthwashes, one after another in three treatment periods. The sequence of mouthwash allocation to the treatment groups was done according to 3 × 3 × 3 Latin square crossover design. Latin square provides uniform crossover designs so that each treatment occurs only once within each sequence and once within each period.^26^ In the Treatment-Sequence Group I, subjects used CHX in the first period, HA in the second period and DW in the third period. Subjects in the Treatment-Sequence Group 2 (n = 11) used HA in the first period, DW in the second period and CHX in the third period. Subjects in the Treatment-Sequence Group 3 used DW in the first period, CHX in the second period and HA in the third period. The randomisation and sequence of mouthwash allocation of the treatment groups are shown in Figure 1.

### Study Design

The present study was designed as a double-blinded, randomised Latin-square controlled, 4-day non-brushing, and crossover experimental study. It was consisted of a pre-experimental period (7 days), first experimental period (4 days), first washout period (10 days), second experimental period (4 days), second washout period (10 days) and third experimental period (4 days). Each experimental period started on Monday mornings and subjects recalled for the measurements on Friday mornings. The total duration of the study was 39 days and it was performed between the dates 9 March and 18 April 2017.

### Interventions

On day 1 of each experimental period, GCF samples were taken from the mesial or distal sites of the first premolar tooth in each quadrant. Full mouth GI was recorded. Professional prophylaxis was then performed to remove all supragingival plaque to establish a baseline of zero plaque scores. The subjects were then asked to stop oral hygiene procedures for the following 4 days. During this period, the only means of oral hygiene that they were allowed was the use of the mouthwash that was allocated to them. Mouthwashes were given in identical opaque bottles. Subjects were asked to rinse with 20 ml in total of the allocated mouthwash to be used twice daily for 30 s at each time, once in the morning after breakfast, once at night before going bed. Subsequent rinsing with water, drinking or eating was not allowed for 30 min after each rinsing time. Rinsing was performed at home without supervision. All instructions were given in detail verbally as well as in written form. During the study period, subjects were asked to follow their normal diet and to avoid the use of chewing gum.^15^ To check for compliance, subjects were asked to note the time of use of mouthwashes onto a calendar record chart and asked to return the bottles that contain mouthwashes. On day 5 of each experimental period, all the measurements including GI and PI and GCF sampling were repeated. Finally, all subjects received a satisfaction questionnaire (SQ) to evaluate their preferences towards the used product. They were questioned about their opinion of appreciation of taste (taste perception), duration and alteration of taste, comfort of use, sensitivity, numbness, and mouth cleanliness. Subjects marked a point on a 10-cm-long uncalibrated horizontal line with the negative extreme response (0) on the left and the positive extreme response (10) on the right end (Visual Analogue Scale) (VAS). All measurements were carried out under the same conditions and were performed by the same examiner (BA) who was blind to the regimens. The washout period was 10 days after each experimental period.^27^ In the wash out period, all subjects returned to their normal oral hygiene methods as were instructed at the beginning of pre-experimental period (no change in individual brushing habits with standardised toothbrush and toothpaste: TePe Supreme, Sensodyne Classic). Only mechanical oral hygiene procedures were allowed during the washout period to eliminate the possible carry-over effects of the mouthwashes.

### Outcome Variables

PI was the primary outcome variable in this study. On day 5 of each experimental period, a disclosing solution (TePe PlaqueSearch) was applied to all teeth except third molars with a cotton swap and the subjects were then asked to rinse with 20 ml of tap water for 15 s. PI was recorded at six sites per tooth (mesiofacial, midfacial, distofacial, mesiolingual, midlingual, distolingual) according to Turesky modification of Quigley Hein Index system (QHI-s).^40^ Scoring criteria with a numerical scale were as follows: 0 = no plaque; 1 = separate flecks of plaque at the cervical margin of the tooth; 2 = a thin continuous band of plaque (up to 1 mm) at the cervical margin of the tooth; 3 = a band of plaque wider than 1 mm at the cervical margin of the tooth; 4 = plaque covering at least one-third but less than two-thirds of the crown of the tooth; 5 = plaque covering two-thirds or more of the crown of the tooth. The scores from the six sites of the tooth were added and divided by six to give the mean value of PI for one tooth. Then, the mean values of all examined teeth were added and divided by the total number of examined teeth to obtain the mean PI score per subject.

GI was assessed by the modified gingival index (MGI), devised by Lobene^19^ on day 1 and on day 5 of the each experimental period, recorded with numbers 0–4, according to the following criteria: 0 = absence of inflammation; 1 = mild inflammation or with slight changes in colour and texture but not in all portions of gingival marginal or papillary; 2 = mild inflammation, such as the preceding criteria, in all portions of gingival marginal or papillary; 3 = moderate, bright surface inflammation, erythema, oedema and/or hypertrophy of gingival marginal or papillary; 4 = severe inflammation: erythema, oedema and/or marginal gingival hypertrophy of the unit or spontaneous bleeding, papillary, congestion or ulceration. The scores from the six sites of the tooth as in PI were added and divided by six to give the mean value of GI for one tooth. Then, the mean values of all examined teeth were added and divided by the total number of examined teeth to obtain the mean GI score per subject.

For the determination of GCF volume, GCF samples were collected with sterile Periopaper strips on day 1 and day 5 of each experimental period. Samples were taken from the mesial or distal site of one premolar tooth from each quadrant. The sites were isolated with cotton rolls and dried with a gentle stream of air. Any visible deposits of supragingival plaque were removed before sampling with Periopaper strips placed carefully into the gingival crevice until mild resistance is felt (1–2 mm into the pocket) and hold in place for 30 s. Strips contaminated by blood or exudate were excluded.^12^ Peritron 8000 was used for the assessment of the GCF volume. Periopaper strips were transferred quickly to the Periotron 8000 device to minimise evaporation errors.^43^ The volume of GCF was immediately recorded, expressed in the measuring device units and followed by calculation of the volume using a standard curve.^17^ SQ form was given to the subjects at the end of each experimental period to evaluate their preferences to the treatment products by using VAS scores. The questions were evaluating the taste perception, duration and alteration of taste, sensitivity, burning sensation, dry mouth, numbness, staining and mouth cleanliness. The list of the complete questions is shown in Figure 2.

**Fig 2:** Questions of satisfaction questionnaire.

**Table:** 

Paraphrase	Complete Questions	VAS scores	
		From –0.0	To –10.0
Taste Perception	How was the taste of the product?	Very bad	Very good
Taste duration	How long did the taste remain?	Very short	Very long
Altered taste	How was the taste of the foods and drinks effected?	Negative change	Positive change
Sensitivity	Did you experience sensitivity?	Not at all	Very much
Burning	Did you experience a burning sensation?	Not at all	Very much
Mouth dryness	Did you experience a mouth dryness?	Not at all	Very much
Numbness	Did you experience a numbness feeling?	Not at all	Very much
Staining	Did you experience a staining on your teeth?	Not at all	Very much
Mouth cleanliness	Did you have the feeling that your teeth were clean after usage of mouthwash?	Not at all	Very much

### Statistical Analysis

Statistical analysis was performed by IBM SPSS Statistics 22 software (Softonic International SA, 1997–2018, Turkey). The compliance of parameters to the normal distribution was evaluated by Shapiro–Wilk test. Intratreatment (days 1–5) comparisons of the parameters with normal distribution evaluated with paired sample t test whereas repeated measures analysis of variance was used for intertreatment comparisons and Bonferroni test waws used as post hoc. Intertreatment comparisons of the parameters without normal distribution evaluated with Friedman test and Wilcoxon signed rank test as post hoc. Statistical significance was set at p < 0.05.

## Results

Thirty-three subjects (15 female, 18 male) aged between 23 and 25 were included in this study. The mean years of ages were 23.29 ± 1.13 The mean PI value was found 1.70 ± 0.27 whereas mean MGI value was 1.37 ± 0.23 at the time of recruitment during the pre-experimental period. All of the randomised subjects, allocated into three different treatment sequences completed the experimental periods and none of them were excluded from the study. A better application of intention to treat approach was achieved and performed since all outcome data were available for all randomised subjects. Table 1 shows the demographics and baseline data of the subjects.

**Table 1:** Demographics and baseline data of the subjects

Number of subjects	33	
Gender	18 F (54.5%) 15 M (45.5%)	
	(Mean)	(SD)
Age (23–25)	23.29	± 1.13
PI	1.70	± 0.27
GI	1.37	± 0.23
PD	< 3 mm	

### Plaque Index

All treatment groups revealed increases in PI values during the experimental periods. On day 5, the mean PI values were 1.64 ± 0.31, 1.81 ± 0.21 and 2.13 ± 0.21 for CHX, HA and DW, respectively, as shown in Table 2a. Intertreatment multiple comparisons of the mean PI values showed a statistically significant difference (p = 0.000) (Table 2a). Further comparisons in pairs revealed statistically significant differences between CHX and HA, CHX and DW, in favour of CHX and between HA and DW in favour of HA (p = 0.048, p = 0.001, p = 0.001, respectively) (Table 2b).

**Table 2a:** Intertreatment multiple comparison of mean PI values on day 5

PI n = 33	CHX	HA	DW	p
	(Mean ± SD)	(Mean ± SD)	(Mean ± SD)	
Day 5	1.64 ± 0.31	1.81 ± 0.21	2.13 ± 0.21	0.000

Repeated measures analysis of variance, p < 0.05.

**Table 2b:** Intertreatment comparisons of the mean PI values in pairs

PI n = 33	CHX vs HA	CHX vs DW	HA vs DW
	p	p	p
Day 5	0.048	0.001	0.001

Bonferroni test, p < 0.05.

### Gingival Index

All treatment groups revealed increases in MGI values during the experimental periods. On day 1, mean MGI values were found 0.55 ± 0.43, 0.58 ± 0.40, 0.51 ± 0.30 for CHX, HA and DW, respectively. No statistically significant difference was detected between the mean MGI values of the treatments on day 1 (p = 0.617). On day 5, mean MGI values were detected as 0.61 ± 0.38, 0.69 ± 0.38, 0.80 ± 0.40, for CHX, HA, and DW, respectively, without any statistically significant differences (p = 0.143). All the treatments showed statistically significant increases from day 1 to day 5 in terms of MGI values (Table 3a). Intertreatment multiple comparisons of the mean changes in MGI values showed a statistically significant difference (p = 0.000). Further comparisons in pairs revealed statistically significant differences between CHX and DW, HA and DW (p = 0.000, p = 0.002, respectively) whereas no statistically significant difference was found between CHX and HA (p = 0.246) (Table 3b).

**Table 3a:** Intertreatment multiple comparisons of mean GI values on day 1, day 5 and of the changes between days 1–5; intratreatment comparisons between days 1 and 5

GI n=33	CHX	HA	DW	p1
	(Mean ± SD)	(Mean ± SD)	(Mean ± SD)	
Day 1	0.55 ± 0.43	0.58 ± 0.40	0.51 ± 0.30	0.617
Day 5	0.61 ± 0.38	0.69 ± 0.38	0.80 ± 0.40	0.143
Change	0.06 ± 0.11	0.11 ± 0.11	0.29 ± 0.25	0.000
p2	0.005	0.000	0.000	

^1^Inter-treatment, repeated measures analysis of variance, p < 0.05; ^2^Intra-treatment, Paired sample t test, p < 0.05.

**Table 3b:** Intertreatment comparisons of the mean GI values in pairs

GI n = 33	CHX vs HA	CHX vs DW	HA vs DW
	p	p	p
Change	0.246	0.000	0.002

Bonferroni test, p < 0.05.

### GCF Volume

The mean values of GCF volume on day 1 and day 5 were detected as 0.58 ± 0.13 and 0.77 ± 0.15 for CHX, 0.61 ± 0.29 and 0.79 ± 0.34 for HA, 0.65 ± 0.27 and 0.91 ± 0.27 for DW, respectively. The changes in the mean values of GCF between days 1–5 were 0.19 ± 0.12, 0.18 ± 0.19, 0.26 ± 0.21 for CHX, HA and DW, respectively. Intratreatment comparisons revealed statistically significant increases in all treatment products (p = 0.000) (Table 4). Intertreatment multiple comparisons of the mean GCF volume showed no statistically significant differences between the treatment products on day 1, day 5, and also for the changes (p = 0.374, p = 0.056, p = 0.186, respectively).

**Table 4:** Intertreatment multiple comparisons of mean GCF volume on day 1, day 5 and of the changes between days 1 and 5; intratreatment comparisons between days 1 and 5

GCF n = 33	CHX	HA	DW	p1
	(Mean ± SD)	(Mean ± SD)	(Mean ± SD)	
Day 1	0.58 ± 0.13	0.61 ± 0.29	0.65 ± 0.27	0.374
Day 5	0.77 ± 0.15	0.79 ± 0.34	0.91 ± 0.27	0.056
Change	0.19 ± 0.12	0.18 ± 0.19	0.26 ± 0.21	0.186
p2	0.000	0.000	0.000	

^1^Intertreatment; repeated measures analysis of variance, p < 0.05; ^2^Intratreatment; Paired sample t test, p < 0.05.

### Satisfaction Questionnaire Responses

Subjects completed SQ after each experimental period and Table 5a shows the mean VAS scores of the subject’s appreciations for the treatment mouthwashes. Multiple comparisons of all satisfaction parameters in SQ revealed statistically significant differences between the treatment mouthwashes for all questions. Statistically significant differences were detected when the comparisons of these parameters performed in pairs (Table 5b), favouring HA in all of them, except for the taste duration and the mouth cleanliness. Furthermore, HA was ranked as the best by 26 out of 33 subjects, for the question of ‘overall first choice’ at the end of all experimental periods.

**Table 5a:** Multiple comparison of the mean VAS scores and preference of the subjects for the overall first choice

Paraphrase	CHX	HA	DW	p
Taste perception	3.33 ± 2.56	6.01 ± 1.95	5.09 ± 2.28	0.002
Taste duration	6.67 ± 1.91	5.70 ± 1.35	0.42 ± 1.25	0.000
Altered taste	6.82 ± 3.07	4.33 ± 1.71	0.12 ± 0.54	0.000
Sensitivity	1.82 ± 3.03	0.79 ± 1.34	0.12 ± 0.41	0.039
Burning	3.82 ± 3.51	1.15 ± 1.58	0 ± 0	0.000
Mouth dryness	2.06 ± 3.62	0.97 ± 1.42	0 ± 0	0.000
Numbness	3.18 ± 3.18	1.21 ± 1.34	0 ± 0	0.000
Staining	2.36 ± 2.61	0.27 ± 0.72	0.02 ± 0.01	0.003
Mouth cleanliness	5.55 ± 2.15	4.36 ± 1.83	0 ± 0	0.000
Overall first choice	6	26	1	

Friedman test, p < 0.05.

**Table 5b:** Intertreatment comparisons of the mean VAS scores in pairs

Paraphrase	CHX vs HA	CHX vs DW	HA vs DW
	p	p	p
Taste perception	0.016	0.023	0.739
Taste duration	0.032	0.000	0.000
Altered taste	0.000	0.000	0.000
Sensitivity	0.101	0.005	0.009
Burning	0.001	0.000	0.001
Mouth dryness	0.001	0.010	0.000
Numbness	0.000	0.000	0.000
Staining	0.008	0.010	0.000
Mouth cleanliness	0.629	0.000	0.713

Wilcoxon sign test, p < 0.05.

## Discussion

This study was designed as a randomised, double-blinded, crossover, 4-day non-brushing clinical study. This study design was suggested by Addy et al^1^ and have the intention to detect the effect of antimicrobial products on new dental plaque formation in the absence of mechanical oral hygiene procedures. It consisted of the use of the tested products by the same subject during a 4-day period when all mechanical oral hygiene procedures were stopped. Randomised controlled studies provide a higher level of evidence for chemotherapeutic agents used for chemical plaque control.^28^ The non-brushing model is the first step to evaluate the plaque inhibitory effects of the agents in vivo, which will give the chance to continue with further long-term study designs using detailed inflammatory and microbial parameters.^21^ Crossover design, by using the same subjects as their own controls for comparing the different treatment products, was selected for reducing the variation to eliminate interindividual differences. In this way, the same subject was used more than once, thereby reducing the sample size required to demonstrate a statistically significant difference between the test and the control treatment products.^6^ On the other hand, crossover study design has the disadvantage of a carry-over effect. The effect of an active ingredient of a treatment product, for example, CHX, may be carried to the following treatment period that may affect the results of the comparisons. Therefore, the washout periods bear clinical importance. To avoid carry-over effects in this study, the subjects were entered to a washout period for 10 days. Although some of the studies used shorter or longer washout periods other than 10 days,^30,37,42^ Newcombe et al^27^ have reported that 10 days washout period is convenient to eliminate the residual effects of CHX from the tissues. Furthermore, the possible carry-over effect of CHX usage in the first period was discarded by a statistical analysis confirming the similarity of the scores of GI and GCF volume at the start of the following study periods evaluating the other treatment mouthwashes. This study also had a double-blind design as neither the volunteers nor the examiner were aware of the composition of the mouthwashes to avoid bias.

PI was used as the primary outcome variable and the QHI-s modified by Turesky et al^40^ was selected for the evaluation of the PI score. Although cervical plaque assessment with this index has a larger validity than its proximal measures,^8^ it is the most commonly used index in non-brushing models due to easy evaluation for disclosed plaque^12,15,21,28,32^ PI system by Sillness and Löe^38^ differentiates the absence or presence of the plaque that is either detectable by a dental probe or visible by the naked eye in different extent around the gingival margin, in which plaque is partially destroyed by the dental probe that is run along the gingival crevice and therefore, further plaque assessment can be impaired. QHI-s was chosen for a more proper assessment of the plaque accumulation without the aforementioned-disadvantages in the present study. PI results obtained by CHX are in the expected range for its activity with statistically significant inhibition on PI scores. Although CHX was found better than HA, HA also exhibited high antiplaque activity comparable to CHX (p = 0.048). This antiplaque activity can be explained by the fact that HA contains xylitol as a preservative ingredient, and xylitol itself has an antiplaque effect.^18,24^ Due to lack of statistically significant data in the literature regarding the usage of HA on the effect of plaque formation, it is not possible to compare our results with the other studies. Only one study, performed by Rodrigues et al,^32^ evaluated the plaque inhibitory effect of HA in comparison with CHX and DW in a 4-day plaque regrowth model with a parallel design. According to the results of that trial, HA showed similar effects to CHX on the PI parameter.

GI by Löe and Sillness^22^ is based on clinical symptoms of inflammation such as gingival colour, contour, marginal bleeding upon dental probe contact and extent of gingival involvement. Bleeding on probing on the other hand, as a relatively objective marker of gingival inflammation, can cause gingival trauma and increase the bleeding after provocation by probing.^21^ Furthermore, bleeding sites can be obscured by blood oozing from previously probed areas to the adjacent tooth surfaces that makes the assessment more difficult.^21^ There is no evidence that invasive indices are truly objective.^21^ In the present study, gingival inflammation was evaluated on the marginal and papillary gingival units by using MGI by Lobene et al^19^ which eliminated the bleeding component and increased the sensitivity at the low-end of the scoring scale. However, the clinical detection of the early inflammatory changes on a 4-day non-brushing model can be considered as questionable due to the possible examiner bias at a known time-point of examination as in the present study.^31^ Therefore, GI was not evaluated as a primary outcome measure, its utility was mainly to assure the healthy gingival conditions at the start of every study period as stated and discussed in Herrera et al 2005.^15^ By using MGI, early inflammatory changes were found much more obvious in DW after 4 days compared to HA and CHX. There was no statistically significant difference between the CHX and HA, which is in accordance with the PI results. It is important to consider and point out the anti-inflammatory and antimicrobial effects of HA. HA has been proven to have long-term anti-inflammatory action, showing a decrease in the amount of plaque-induced gingivitis.^7^ Anti-inflammatory action of HA is thought to be due to its scavenging action on matrix metalloproteinases and prostaglandins which are the mediators of the inflammation.^14,16,33^ The antimicrobial effect on the other side is suggested as bacteriostatic influence in recent systemic reviews and original studies.^7,32,39^ The results of the present study with respect to the MGI values are in accordance with the study by Rodrigues et al.^32^ They did not also find any statistical statistically significant differences in terms of gingival inflammation between HA and CHX mouthwashes revealing their similar efficacy.

Gingival inflammation was further assessed by the quantitative evaluation of GCF volume through using a calibrated electronic device. GCF is a serum transudate of clinically normal periodontal tissues that becomes an inflammatory exudate when the disease is clinically detectable.^14,17,43^ The collection and evaluation of GCF samples before and after the usage of treatment mouthwashes in this study aimed to support the clinical findings of early inflammatory changes of the gingival tissues during the 4-day non-brushing period. The results of the present study showed statistically significant intratreatment GCF volume changes between days 1 and 5 in all treatment-sequence groups. The differences between the increased GCF volumes on day 5 did not reach a statistical significance, however, DW revealed a higher value than CHX and HA (0.77 ± 0.15, 0.79 ± 0.34, 0.91 ± 0.27, respectively). There are inconsistent findings in the literature whether this parameter is a reliable marker of early inflammation. Some studies found a positive correlation between GCF volume and early clinical signs of inflammation, whereas others did not.^17,20^ However, GCF volume increases in our treatment groups were obviously in accordance with the clinical findings.

VAS scale was used for the evaluation of the patient satisfaction. Taste perception, duration of the taste, alteration of taste, sensitivity, burning sensation, dry mouth, numbness, staining and mouth cleanliness were questioned. Statistically significant differences were found for every question, favouring HA compared to CHX and DW, except for the taste duration and mouth cleanliness. At the end of all experimental periods, the patients were asked for their first preference among the treatment mouthwashes and HA was ranked as the best by 26 out of 33 subjects.

## Conclusion

Within the limits of this study, HA revealed an almost similar plaque inhibitory effect to CHX. Also, early gingival inflammatory changes were found similar, supporting the anti-inflammatory properties of HA. For HA, subjects reported significantly better taste, less sensitivity, taste alteration, burning sensation, mouth dryness, numbness perception and staining as compared to CHX except for the mouth cleanliness and duration of taste. Based on the present findings of this 4-day non-brushing clinical trial, future studies are warranted to further evaluate the long-term antiplaque as well as anti-inflammatory and antimicrobial effects of HA.
